# Development of a Liquid-Phased Probe Array for Upland Cotton and Its Application in Cultivar Identification

**DOI:** 10.3390/genes17010008

**Published:** 2025-12-21

**Authors:** Haiyan Tian, Yongping Zhou, Yongqiang Wang, Mengzhe Li, Guiyuan Zhao, Haiying Du, Jianguang Liu, Zhao Geng

**Affiliations:** Key Laboratory of Cotton Biology and Genetic Breeding in Huanghuaihai Semiarid Area, Ministry of Agriculture and Rural Affairs, Institute of Cotton, Hebei Academy of Agriculture and Forestry Sciences, Shijiazhuang 050051, China

**Keywords:** cotton, in-solution probe, genotyping by target sequencing, cultivar identification

## Abstract

Single-nucleotide polymorphism (SNP) genotyping arrays are important tools for crop genetic research. Addressing the current issues of insufficient accuracy in upland cotton cultivar identification and difficulties in distinguishing closely related germplasm and hybrids, developing an SNP array enabling rapid and accurate cotton cultivar identification and applicable to molecular breeding is a key demand in cotton cultivar identification and genetic breeding. This study aims to develop a low-cost and high-precision SNP array for upland cotton (*Gossypium hirsutum* L.) based on Genotyping by Target Sequencing (GBTS) technology. The array will integrate high accuracy in cultivar identification with applicability to molecular breeding, and this study further aims to clarify its application in cultivar identification. The Cotton 13K SNP array contains 13,571 high-quality SNP loci, including 8658 polymorphic sites derived from resequencing data and 4913 functional loci linked to key agronomic traits. All these loci are relatively evenly distributed across the genome. Genotyping 219 upland cotton cultivars/lines accurately clustered them into four genetic subgroups (K = 4), which closely matched their breeding institutions and geographical origins. Analysis of 44 experimental cotton materials (including sister lines and backcross materials) established a genetic similarity threshold of ≥90% for effectively distinguishing closely related germplasm. Comparative analysis of 38 F_1_ hybrids and conventional cotton cultivars demonstrated that the average heterozygosity (Het) of hybrids (16.01%) was significantly higher than that of conventional cultivars (5.52%, *p* < 0.001). A preliminary threshold of Het ≥ 10% was identified for accurate discrimination of cotton hybrids. In conclusion, the Cotton 13K SNP array is a robust tool for population genetic analysis, discrimination of closely related cultivars, and hybrid identification. It also facilitates key molecular breeding steps, including parental evaluation, backcross monitoring, and marker-assisted selection (MAS). Its integration into breeding pipelines is expected to accelerate the development of new cotton varieties.

## 1. Introduction

Cotton (*Gossypium hirsutum* L.) is among the most important economic crops in the world, and accurate varietal identification is crucial for preserving intellectual property related to breeding, controlling seed quality, and managing germplasm resources [1]. The narrow genetic base of contemporary breeding materials has resulted in increasingly similar genetic backgrounds among newly released varieties. Conventional identification methods, which rely on morphological and biochemical markers, are often susceptible to environmental influences, leading to low discriminatory power [2,3]. In contrast, early molecular marker systems (e.g., RFLP and SSR) have several limitations. These include low throughput, limited marker numbers, uneven genomic distribution, insufficient polymorphism, incomplete genome coverage, and challenges in integrating and comparing data across different laboratories [4,5].

Due to their advantages of genome-wide distribution, high density, and compatibility with automated detection, SNP markers have emerged as a crucial tool for variety identification and genetic analysis [6,7,8,9]. In cotton research, high-density SNP array technology has undergone rapid development. Several solid-state SNP arrays, including CottonSNP 63 K [10] and CottonSNP 80 K [11], have been developed and widely adopted. This higher marker density significantly improves the efficiency and accuracy of intraspecific polymorphism detection. These high-density SNP arrays exhibit strong utility and reliability in a variety of genetic studies [12,13,14]. However, high-density solid-phase SNP arrays have a relatively high per-unit cost. This restricts their use in large-scale germplasm identification and conventional breeding—two areas where low-cost solutions are highly needed.

Genotyping by target sequencing (GBTS), a representative liquid-phase chip technology, has attracted widespread attention because of its high flexibility and cost-effectiveness. It has broad applications in germplasm evaluation, gene mapping and cloning, genetic map construction, marker-assisted selection (MAS), and variety protection [15]. Additionally, it has been successfully applied in several crops, including wheat [16], maize [17], soybean [18], and rapeseed [19]. In cotton research, the “ZJU CottonSNP40 K” chip was developed for cotton using GBTS technology [20]. Compared with solid-phase arrays, although this array offers flexibility, it still lacks a cost advantage in large-scale cultivar identification and marker-assisted selection (MAS).

Current methods for identifying upland cotton (*G. hirsutum* L.) varieties face challenges such as insufficient accuracy and difficulty distinguishing closely related germplasms, while existing ultrahigh-density genotyping arrays remain costly. To address these issues, this study aims to develop a cost-effective, high-accuracy liquid-phase SNP chip for upland cotton. This will be achieved by integrating whole-genome resequencing data from representative upland cotton germplasms with genome-wide association study (GWAS) loci associated with key agronomic traits. This chip will provide an efficient technical tool for cotton variety identification and genetic improvement.

## 2. Materials and Methods

### 2.1. Plant Materials

A subset of the liquid chip markers developed in this study was derived from whole-genome resequencing data of 100 *G. hirsutum* L. accessions (Supplementary Table S1). This population exhibits broad genetic and geographical diversity. It includes 35 germplasm accessions and 65 cultivars from three major cotton-growing regions of China: 50 from the Yellow River Valley, 6 from the Yangtze River Valley, and 9 from the Northwest Inland. The materials include both conventional and hybrid cotton varieties, as well as diverse types: high-phenolic, low-phenolic, early-maturing, medium-maturing, and colored. This diversity ensures comprehensive coverage of the markers.

To evaluate the SNP array’s applicability for cotton cultivar identification, we performed genotyping and analysis on three sets of materials. First, 219 cotton varieties were used for population genetic structure analysis (Supplementary Table S2). Second, 44 breeding materials (including sister lines, backcross progenies from the same recurrent parent, and individual plants from the same progeny row) were used to identify genetically similar materials (Supplementary Table S3). Third, 38 F_1_ hybrids and 38 pure-line cultivars were used for hybrid identification (Supplementary Table S4).

### 2.2. Collection and Screening of Functional SNP Markers in G. hirsutum L.

To increase the application value of liquid-phase chips in molecular breeding, in this study, SNP markers associated with important traits in *G. hirsutum L.* were systematically identified and collected from publications between 2016 and 2023. We conducted a literature retrieval on Google Scholar using the keywords “*Gossypium hirsutum*” and “GWAS”. After manual screening, 69 articles were retained (Supplementary Table S5) [7,11,13,14,21,22,23,24,25,26,27,28,29,30,31,32,33,34,35,36,37,38,39,40,41,42,43,44,45,46,47,48,49,50,51,52,53,54,55,56,57,58,59,60,61,62,63,64,65,66,67,68,69,70,71,72,73,74,75,76,77,78,79,80,81,82,83,84,85], resulting in the collection of 25,492 SNP loci. These loci are associated with key cotton traits, including yield, fiber quality, agronomic traits, biotic stress resistance, and abiotic stress tolerance.

100 bp flanking sequences of candidate SNPs were aligned to the reference genome (*G. hirsutum* TM-1_ICR) using BLAST (v2.1.0), and probe design feasibility was evaluated based on the following criteria: (1) single-copy loci (sequence identity > 60%, alignment length > 60%); (2) GC content of 40–60%; (3) no repeat sequences or additional N bases in flanking regions; and (4) no InDels > 5 bp within flanking regions [86,87].

### 2.3. SNP Detection and Screening of Whole-Genome Resequencing Data

**Figure 1:**
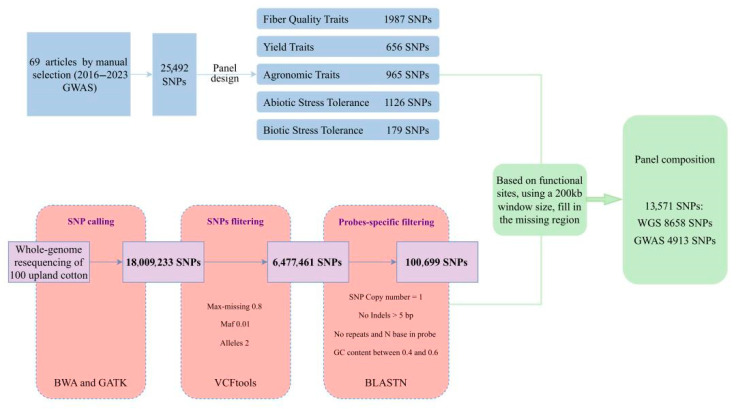
The SNP selection process for the 13K liquid-phase array of *Gossypium hirsutum* L.

The cotton SNP array development workflow is detailed in Figure 1. Genomic DNA was extracted from samples and quality-checked; qualified samples were submitted to Annoroad Gene Technology (Beijing) Co., Ltd. (Beijing, China) for 10× genome coverage sequencing. Raw data were processed to generate high-quality clean reads.

Clean reads were aligned to the reference genome using BWA (v0.7.17) [88] with default parameters. Following coordinate sorting and duplicate marking, GATK (v4.0) [89] was used to call variants per sample. Raw variants were filtered by: QUAL < 30, QD < 2.0, FS > 60.0, and MQ < 40.0.

Filtered SNPs underwent further strict filtering using VCFtools (V4.1) (parameters: —max-missing 0.8, —maf 0.01, —min-alleles 2, —max-alleles 2), followed by screening against the probe design feasibility criteria in Section 2.2. Final SNPs were functionally annotated using SnpEff (V5.2) [90] to identify variants with potential functional impacts and guide subsequent locus prioritization.

### 2.4. SNP Marker Integration

Single-nucleotide polymorphism (SNP) markers from GWAS and whole-genome resequencing data were integrated. Upland cotton genome characteristics and target probe density were considered. A 200 kb window size was determined through calculation. Each window contains at least one high-confidence marker. This achieves uniform distribution of markers across the entire genome. During window sliding, the “GWAS-associated locus priority” principle applied: windows with GWAS-associated loci were directly retained; otherwise, resequencing-derived SNPs were filtered. Resequencing-derived SNPs were prioritized as follows: first, loci optimal for probe design; among these, SNPs with strong functional impacts (e.g., non-synonymous mutations, start/stop codon variations) were preferred. Multiple qualifying SNPs were randomly selected one.

### 2.5. Liquid-Phase Hybridization Probe Capture

Liquid-Phase Hybridization Probe Capture was carried out following the detailed protocol reported by Yu et al. [91]. This study described the development of a 50 K SNP array for eggplant (*Solanum melongena* L.), and its established capture protocol was adopted herein.

### 2.6. Phylogenetic and Population Structure Analysis

Population structure analysis was performed using ADMIXTURE v1.3.0 [92]. The optimal K value (K = 2–10) was determined through cross-validation, with K = 4 selected as the best population division scheme. For principal component analysis (PCA), PLINK v1.90 [93] was used to calculate the first three principal components (PC1–PC3) of SNP data from the array loci, revealing genetic differentiation patterns within and among populations. Based on the p-distance genetic distance between individuals, a neighbor-joining phylogenetic tree was constructed using TreeBest v1.9.2 [88], validated by 1000 bootstrap replicates.

### 2.7. Preliminary Application of the SNP Liquid-Phase Chip in Variety Identification

#### 2.7.1. Distinction of Cotton Germplasm with Close Genetic Relationships 

Based on the obtained SNP genotyping data, the genetic similarity between all pairwise combinations of samples was calculated. Genetic similarity was defined as the proportion of SNP loci with identical genotypes between two samples relative to the total number of SNPs analyzed.

#### 2.7.2. Cotton Hybrid Identification

On the basis of the obtained SNP genotyping data, the individual heterozygosity of each sample was calculated. Individual heterozygosity was defined as the proportion of heterozygous loci in a sample relative to the total number of SNPs analyzed.

## 3. Results

### 3.1. SNP Selection and Array Design

A total of 25,492 SNP loci associated with target traits were manually curated from the literature. Following the evaluation of probe design feasibility, 4913 SNPs were ultimately selected for chip construction, including those associated with yield traits (656), fiber quality (1987), agronomic traits (965), abiotic stress (1126), and biotic stress (179) (Supplementary Table S6).

Using whole-genome resequencing data from 100 *G. hirsutum* L. germplasm accessions, we identified 18,009,233 high-quality SNPs for downstream analyses. These SNPs were stringently filtered using VCFtools, resulting in 6,477,461 SNPs. Finally, 100,699 SNPs were selected on the basis of the probe design criteria.

We adopted a functional site prioritization strategy, integrating 4913 functional SNPs from the GWAS and 8658 SNPs generated from resequencing, ultimately constructing a chip containing 13,571 representative SNPs (Supplementary Table S7).

### 3.2. Characteristics of the Cotton 13K SNP Liquid-Phase Chip

**Figure 2:**
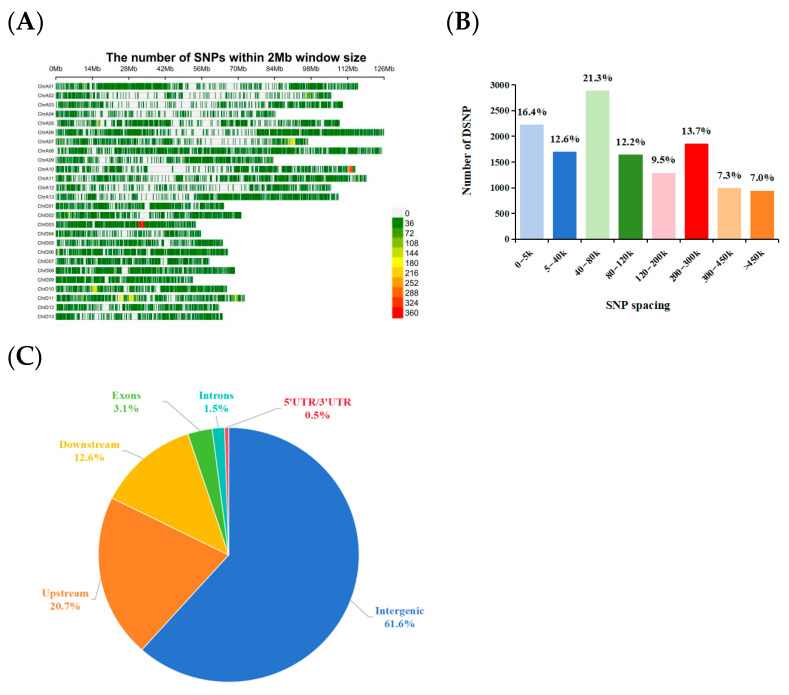
Characteristics of the Cotton 13K array. (**A**) Distribution of 13,571 SNPs across 26 chromosomes. The window size was 2 Mb. (**B**) The distribution of spacing between adjacent SNP loci in the genome. (**C**) The distribution of SNPs in different genomic regions.

The 13,571 SNPs were relatively evenly distributed across all 26 chromosomes, with an average density of 6.10 SNPs/Mb (Figure 2A, Table 1). Statistical analysis revealed that the number of SNPs per chromosome ranged from 230 to 906, with the SNP density varying between 2.72 and 14.83 SNPs/Mb among chromosomes. Among these, chromosome D03 had the highest SNP density (14.83 SNPs/Mb), followed by D11 (12.49 SNPs/Mb), while A04 had the lowest density (2.72 SNPs/Mb). Physical interval analysis revealed that the average distance between adjacent SNP pairs was 164.06 kb. The frequency distribution of SNP spacing was as follows: 29.0% (3925 SNPs) were spaced within 40 kb, 21.3% (2887) were between 40 and 80 kb, 12.2% (1646) were between 80 and 120 kb, 9.5% (1293) were between 120 and 200 kb, 13.7% (1858) were between 200 and 300 kb, and 14.3% (1936) exceeded 300 kb (Figure 2B).

Functional annotation of the SNPs revealed that the majority were located in intergenic regions, followed by upstream and downstream gene regions (Figure 2C).

Specifically, among the 13,571 SNPs, 8361 SNPs (61.6%)were located in intergenic regions, 2814 SNPs (20.7%) were located in upstream regions of genes, and 1707 (12.6%) were located in downstream regions of genes. Furthermore, the array included 414, 205, and 70 SNPs in the exons, introns, and 5′ and 3′ untranslated regions, respectively.

### 3.3. Evaluation of Chip Capture Efficiency

To better assess the capture efficiency of the cotton liquid-phase chip, we performed target region capture and bioinformatic analysis on 12 representative cotton varieties (Table 2). The results revealed high-quality sequencing data across all the samples, with average Q20 and Q30 values of 97.41% and 94.61%, respectively. Furthermore, alignment analysis showed an average mapping efficiency of 98.02% (range: 91.70–99.39%). This confirms that most sequencing reads were accurately mapped to the reference genome. In terms of capture performance, the on-target rate averaged 61.32% (range: 61.03–61.69%), indicating the high capture specificity of the probe panel. Coverage analysis demonstrated uniform and reliable sequencing depth across the target regions, with a mean depth of 285.17× (range: 215×–347×). Furthermore, high coverage completeness was achieved, as reflected by an average coverage rate of 98.44%. This confirms effective coverage of most target sites and supports high-confidence SNP calling and genotyping. These results demonstrated the good repeatability and accuracy of the 10K liquid-phase chip.

### 3.4. Population Structure Analysis Based on the Cotton SNP Liquid-Phase Chip

**Figure 3:**
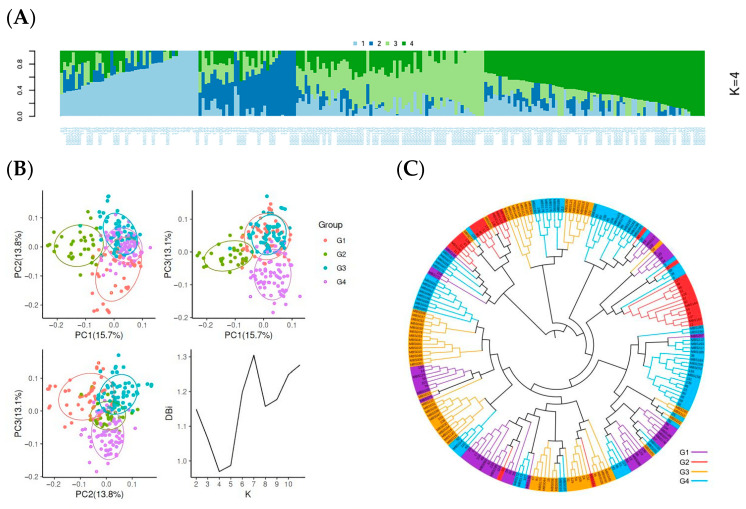
Population structure of 219 cotton materials based on the Cotton 13K SNP array. (**A**) Population structure analysis of the 219 cotton materials; different colors represent different groups. (**B**) Principal component analysis (PCA) of the materials; different colors represent different groups. (**C**) Phylogenetic tree analysis of the 219 cotton materials; different colors represent different groups.

To evaluate the application potential of this chip, 219 cotton materials (Supplementary Table S2) were selected for genotyping analysis. The test materials included commercial varieties, germplasm resources, and self-bred lines. Population structure analysis was performed on all the samples on the basis of the chip genotyping data. The optimal number of genetic populations was determined to be K = 4 via cross-validation, and all the samples were divided into four subpopulations (Figure 3A). Analysis of material composition within the population revealed that the materials in each subpopulation clearly clustered by breeding unit or geographical origin, indicating a high correlation between population division and the genetic background of the materials. Further validation of the above classification results was conducted using principal component analysis (PCA) (Figure 3B). The PCA results revealed that the 219 materials formed four distinct clusters in two-dimensional space, corresponding to different variety series, indicating that the chip has high resolving power. To analyze population genetic relationships, a phylogenetic tree was constructed using TreeBest (v.1.9.2) (Figure 3C). Its topological structure significantly supported the division of the four subpopulations (G1–G4) and exhibited an obvious clustering pattern by breeding unit and geographical origin. Specifically, G1 included 45 materials, mainly containing Guoxin series and Jishi series varieties (e.g., Guoxinmian 25, Guoxinmian 31, Jishi 813); G2 had 30 materials, dominated by Nongdamian series and Hengmian series varieties (e.g., Ji’nongdamian 23, Hengmian 1670); G3 comprised 67 materials, with key components including new lines, hybrid combinations from the chip development unit, and Jimian series varieties (e.g., Ji 228, Jimian 632, Jimian 128); and G4 contained 77 materials, mainly including Hanmian series, Lumian series, and Zhongmian series varieties (e.g., Han 3026, Lumianyan 21, Zhongmian 9001).

These results indicate that the cotton 13K SNP chip can efficiently and accurately resolve the genetic structure of a cotton population and clearly distinguish materials with similar genetic backgrounds. The subpopulation classification revealed by the chip genotyping results is highly consistent with the breeding units and geographical origins, confirming its good application value in the genetic diversity analysis, population genetic structure analysis, and germplasm resource identification of cotton.

### 3.5. The Application of the 13 K SNP Liquid Chip in Cotton Variety Identification

#### 3.5.1. Discrimination of Closely Related Cotton Germplasm

**Figure 4:**
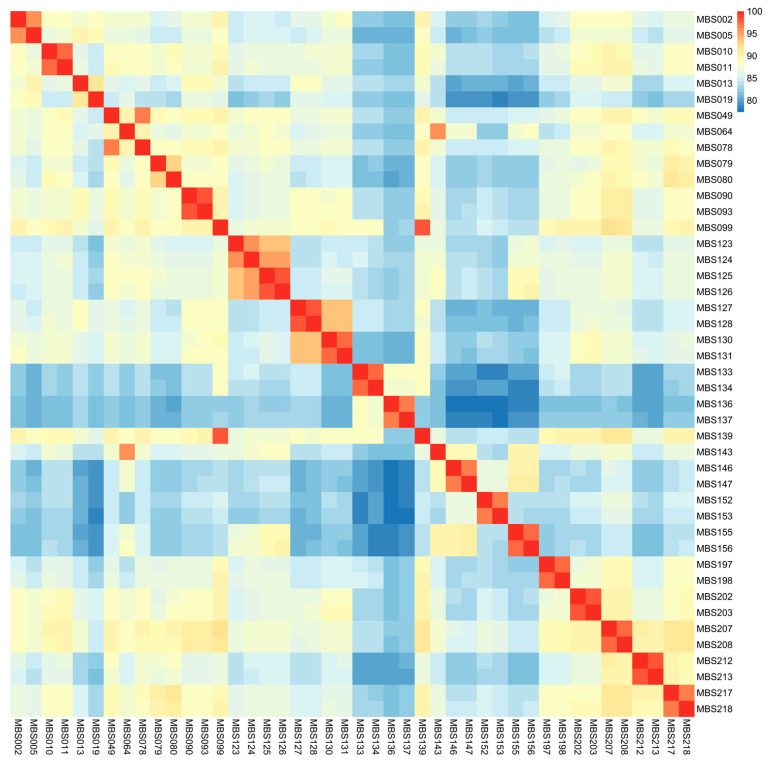
Heatmap of genetic similarity among 44 cotton test materials.

SNP genotyping was performed on 44 cotton test materials (Supplementary Table S3). Based on pairwise genetic similarity analysis, the genetic similarity among materials ranged from 77.38% to 98.49%, indicating significant genetic differentiation within the population (Figure 4).

We conducted further analysis on sample groups with highly similar genetic backgrounds. These groups included sister lines, backcross individuals with the same recurrent parent, and different individuals from the same plant row. Their genetic similarity ranged from 91.33% to 98.49%—a relatively high level. This was significantly different from the genetic similarity of materials with distant genetic relationships.

Specifically, in different backcross materials sharing the same recurrent parent, genetic similarity was positively correlated with the number of backcross generations. Three backcross combinations (13/19, 2/5, and 10/11) had backcross-selfing generations of BC_3_F_7_, BC_3_F_8_, and BC_4_F_7_, respectively, with corresponding genetic similarities of 91.33%, 95.66%, and 97.21%, respectively. These results indicate that as the number of backcross generations increased, the genetic background of the materials became increasingly consistent. Among sister line materials, the 49/78, 79/80, and 123/125 combinations had genetic similarities of 96.39%, 92.90%, and 93.53%, respectively. By contrast, combinations of different individuals from the same plant row—such as 125/126, 127/128, and 146/147—showed higher genetic consistency. Their respective genetic similarities reached 98.03%, 98.05%, and 96.26%.

On the basis of the results of the analysis of the 44 cotton test materials, this study preliminarily revealed that a genetic similarity of 90% could be used as a threshold to identify materials with similar genetic origins, such as sister lines, the same backcross population, or materials derived from the same plant row.

The results demonstrate the developed SNP chip has high polymorphism and resolution. It can effectively distinguish materials with highly similar genetic backgrounds. Additionally, it provides a reliable basis for identifying cotton materials with unknown genetic relationships—such as potential sister lines.

#### 3.5.2. Identification of Hybrids

To verify the independently developed SNP chip’s application potential in hybrid authenticity identification, this study genotyped 38 cotton F_1_ hybrids and 38 pure-line cultivars using the chip. The analysis focused on individual heterozygosity (Het) characteristics (Supplementary Table S8). The genotyping results revealed that the average Het of the F_1_ hybrid population was 16.01% (distribution range: 10.51–22.08%), while the average Het of the pure-line cultivar population was 5.52% (distribution range: 3.99–7.34%). The Het difference between the two groups was extremely significant (*p* < 0.001), and their distribution intervals were completely nonoverlapping. Specifically, the minimum Het of hybrids was 10.51%, while the maximum Het of pure-line cultivars was 7.34%. On the basis of this distribution gap, “Het ≥ 10%” was proposed as the determination threshold for F_1_ hybrids. Although F_1_ hybrids showed large Het variation (10.51–22.08%), all of them were stably above the 10% threshold. This confirms the standard’s reliability in practical applications.

## 4. Discussion

In this study, a 13 K SNP liquid-phase chip applicable to upland cotton was successfully developed. This chip integrates 4913 functional loci associated with important traits and 8658 genome-wide representative loci. It thus achieves a good balance among functional locus orientation, genome coverage, and cost-effectiveness. This design concept aligns with functional marker integration schemes—such as the maize 50K [90] and rapeseed 54K GBTS chips [19]. It reflects the development trend of modern molecular breeding chips: functionalization, practicalization, and cost reduction.

The 13,571 SNPs included in this chip exhibit significant differences in distribution density across chromosomes (range: 2.72–14.83 SNPs/Mb). This variation stems primarily from the chip’s “functional locus priority” design strategy, leading to significantly higher SNP density in functional marker-enriched chromosomal regions (e.g., chromosome D03, the highest at 14.83 SNPs/Mb). This function-oriented differential distribution feature differs markedly from the chip design strategies adopted in crops such as maize [90] and rapeseed [19], which are centered on uniform coverage or resequencing-based filling. It confers distinct advantages for subsequent assisted breeding applications, enhancing the accuracy and efficiency of marker-assisted selection (MAS).

Results of population genetic analysis fully validated the chip’s ability to resolve complex population structures and successfully distinguished varieties (lines) with highly similar genetic backgrounds. These results not only effectively address the limitations of traditional morphological and low-throughput molecular marker methods in distinguishing closely related materials but also objectively reflect the pattern of genetic differentiation among geographical regions and breeding institutions within the Chinese cotton breeding system. These findings align with those reported by Wei, J. [94], Panhong, D. et al. [95], In their studies on the genetic diversity of Chinese upland cotton varieties, these researchers reported a significant association between cultivar relatedness and geographical origin. This confirms the chip’s high reliability and broad application potential in cotton population genetic research.

Notably, although each subpopulation is primarily dominated by varieties from specific breeding institutions, individual varieties also appear in other subpopulations. This to some extent reflects germplasm resource exchange among breeding institutions and regions. In terms of variety identification applications, traditional morphological methods are often limited in their ability to distinguish materials with genetic similarity higher than 90%. The 13 K liquid-phase chip developed in this study significantly improved molecular resolution and can effectively distinguish cotton experimental materials with genetic similarity as high as 98.49%. Based on studies of 44 breeding materials, we preliminarily propose 90% genetic similarity as a threshold for distinguishing these highly homogeneous breeding materials. This provides a powerful technical solution: it addresses the challenges of high genetic similarity and ineffective differentiation among new varieties, which stem from the relatively narrow genetic basis of current breeding parents. The chip’s excellent discriminatory ability is presumably due to the “functional locus priority” principle adopted during its design. This principle results in significantly higher SNP density in certain chromosomal regions compared to the genome-wide average. This local high-density characteristic can more sensitively capture and amplify subtle genetic differences between materials (e.g., sister lines) from a functional perspective, thereby enabling accurate identification.

The identification of cotton hybrids is critical for ensuring production efficiency and seed industry integrity. On the basis of an independently developed SNP array, in this study, a heterozygosity (Het)-based identification system was established, and a Het threshold of ≥10% for discriminating F_1_ hybrids was proposed. This method is suitable for rapid high-throughput detection in seed quality monitoring and market circulation. Variation in heterozygosity was observed among different F_1_ hybrids (range: 10.51–22.08%), which was closely associated with parental genetic divergence. For example, the self-bred hybrid combination MBS31 (high quality × high yield), with significant phenotypic differences, exhibited a Het of 20.38%, whereas the phenotypically similar combination MBS150 (high quality and yield × high quality and yield) had a Het of 11.74%. All tested self-bred combinations and commercial hybrids consistently displayed Het values above 10%, indicating the broad applicability of this threshold; however, its validation across more diverse materials requires further investigation. To improve the reliability of identification, follow-up studies will incorporate the detection of parent-specific SNP markers on the basis of preliminary Het screening. This approach will first confirm whether a sample is a hybrid based on heterozygosity. It will then verify if it is the target F_1_ using specific loci, achieving integrated authenticity identification and parental traceability. This ultimately enhances the specificity and reliability of the results significantly.

Compared with existing cotton SNP platforms (e.g., CottonSNP 63 K, CottonSNP 80 K, ZJU CottonSNP40K), the 13 K SNP liquid-phase chip developed in this study has lower density. However, its design offers two distinct advantages, achieving differentiation from current platforms. Firstly, it leverages the unique dynamic updating capability of liquid-phase platforms. This enables real-time integration of newly discovered functional loci, addressing the technical bottleneck of difficult iteration faced by traditional solid-phase platforms like CottonSNP63K.Secondly, its streamlined design of “functional targeting + moderate coverage” confers significant cost benefits. It is more applicable in large-scale scenarios, such as variety identification and marker-assisted selection (MAS). This effectively resolves the limited large-scale application of existing ultra-high-density chips due to high costs.

Although this chip performs excellently in variety identification and population structure analysis, it still has limitations: Given the high genetic diversity of cotton germplasm, further validation of the universality of chip loci and genotyping accuracy is required using materials from broader sources. Future optimization can focus on two aspects: (1) Integrate whole-genome sequencing data from more core germplasm, dynamically update the locus library, and improve detection accuracy and applicability; (2) Track advances in cotton functional genomics and molecular breeding, promptly incorporate new functional loci associated with important agronomic traits, and enhance its application value in molecular design breeding.

## 5. Conclusions

In summary, the 13 K SNP liquid chip developed in this study balances functional orientation and economic feasibility. It has enabled efficient analysis of the population genetic structure of *G. hirsutum* L. and accurate molecular identification of cotton varieties (lines). This chip, which is an efficient, flexible, and cost-controllable genotyping platform, is expected to play a key role at multiple levels: (1) the construction of a standardized DNA fingerprint database for cotton varieties, providing an objective and traceable molecular basis for variety rights protection (e.g., DUS testing); (2) the application to seed quality supervision to quickly identify seed authenticity and purity, crack down on counterfeit and shoddy products, and maintain market order; and (3) the deep integration into the entire process of molecular breeding, including parental genetic evaluation and selection, background selection, tracking the progress of backcross breeding, and marker-assisted selection (MAS) of important functional loci, thereby significantly accelerating the breeding process of new cotton varieties.

## Figures and Tables

**Table 1:** The SNPs number on each chromosome and their distance.

Chromosome	Length (Mb)	Number of SNPs	Density of SNP (SNPs/Mb)	Average Distance (Kb)
A01	115.95	703	6.06	164.94
A02	105.67	346	3.27	305.41
A03	110.12	427	3.88	257.90
A04	84.63	230	2.72	367.95
A05	108.86	604	5.55	180.24
A06	126.11	742	5.88	169.96
A07	96.73	622	6.43	155.51
A08	125.57	807	6.43	155.60
A09	83.56	463	5.54	180.48
A10	114.91	793	6.90	144.91
A11	119.36	644	5.40	185.34
A12	105.78	348	3.29	303.95
A13	108.61	552	5.08	196.75
D01	64.53	412	6.38	156.63
D02	71.14	504	7.08	141.14
D03	53.68	796	14.83	67.44
D04	56.27	242	4.30	232.52
D05	64.11	359	5.60	178.59
D06	66.01	562	8.51	117.46
D07	58.84	366	6.22	160.76
D08	68.68	493	7.18	139.30
D09	52.56	375	7.13	140.16
D10	65.68	599	9.12	109.65
D11	72.55	906	12.49	80.07
D12	62.68	351	5.60	178.57
D13	63.92	325	5.08	196.68

**Table 2:** The 13K liquid-phase chip detection coverage statistics.

Material Name	Mapped (%)	Target Ratio (%)	Average Depth	Coverage Rate (%)	Reads_Num (M)	Data (G)	Q20 (%)	Q30 (%)	GC (%)
Han 5158	99.20	61.69	316	98.51	5.29	1.59	97.46	94.70	40.68
Hanmian 802	99.39	61.64	343	98.75	5.90	1.78	97.38	94.55	40.53
Ji 668	98.98	61.60	287	98.45	4.74	1.43	97.41	94.63	40.85
Jimian 20	99.19	61.51	248	98.34	4.09	1.23	97.37	94.55	40.77
Lumianyan 21	99.07	61.32	332	98.65	5.84	1.76	97.32	94.43	40.50
Ji 228	91.70	61.31	250	98.57	4.56	1.37	97.30	94.37	40.34
Jimian 616	99.33	61.28	297	98.68	5.03	1.52	97.40	94.60	40.58
Guoxinmian 3 Hao	95.55	61.24	239	98.00	3.96	1.19	97.56	94.94	40.94
Kuaiyu 66	96.00	61.09	215	97.74	3.51	1.06	97.69	95.31	40.59
Hanwu 23	99.38	61.07	247	98.41	4.21	1.27	97.44	94.67	40.72
Jifeng 914	99.18	61.05	347	98.78	5.95	1.79	97.29	94.33	40.55
Jimian 298	99.27	61.03	301	98.39	5.06	1.53	97.26	94.26	40.63
Average	98.02	61.32	285.17	98.44	4.85	1.46	97.41	94.61	40.64

## Data Availability

The original contributions presented in this study are included in the article/Supplementary Materials. Further inquiries can be directed to the corresponding author.

## Supplementary Materials

The following supporting information can be downloaded at: https://www.mdpi.com/article/10.3390/genes17010008/s1, Table S1: List of 100 test materials; Table S2: Cotton varieties used for phylogenetic and population structure analysis; Table S3: Cotton accessions used for identification of genetic similarity; Table S4: Cotton Accessions for Hybrid Identification; Table S5: List of 69 references; Table S6: List of 4913 functional SNPs; Table S7: The 13571 SNPs from cotton 13 K SNP array; Table S8: Comparison of heterozygosity between cotton hybrids and pure-line cultivars.
